# Promoter characterization of a *citrus* linalool synthase gene mediating interspecific variation in resistance to a bacterial pathogen

**DOI:** 10.1186/s12870-023-04413-6

**Published:** 2023-08-25

**Authors:** Qiying Wang, Xiaochun Wang, Linhua Huang, Yujiao Cheng, Li Ren, Huayu Yang, Changyong Zhou, Xuefeng Wang, Jun He

**Affiliations:** National Citrus Engineering Research Center, Citrus Research Institute, Southwest University, Chongqing, 400712 China

**Keywords:** Citrus, Canker disease, Linalool, Terpene synthase, Promoter

## Abstract

**Background:**

Terpenoids play essential roles in plant defense against biotic stresses. In *Citrus* species, the monoterpene linalool mediates resistance against citrus canker disease caused by the gram-negative bacteria *Xanthomonas citri* subsp. *citri* (*Xcc*). Previous work had associated linalool contents with resistance; here we characterize transcriptional responses of linalool synthase genes.

**Results:**

Leaf linalool contents are highly variable among different *Citrus* species. “Dongfang” tangerine (*Citrus reticulata*), a species with high linalool levels was more resistant to *Xcc* than “Shatian” pummelo (*C. grandis*) which accumulates only small amounts of linalool. The coding sequences of the major leaf-expressed linalool synthase gene (*STS4*) are highly conserved, while transcript levels differ between the two *Citrus* species. To understand this apparent differential transcription, we isolated the promoters of *STS4* from the two species, fused them to a GUS reporter and expressed them in Arabidopsis. This reporter system revealed that the two promoters have different constitutive activities, mainly in trichomes. Interestingly, both linalool contents and *STS4* transcript levels are insensitive to *Xcc* infestation in citrus plants, but in these transgenic Arabidopsis plants, the promoters are activated by challenge of a bacterial pathogen *Pseudomonas syringae*, as well as wounding and external jasmonic acid treatment.

**Conclusions:**

Our study reveals variation in linalool and resistance to *Xcc* in citrus plants, which may be mediated by different promoter activities of a terpene synthase gene in different *Citrus* species.

**Supplementary Information:**

The online version contains supplementary material available at 10.1186/s12870-023-04413-6.

## Background

Citrus is the largest fruit category in the world. Citrus canker is a devastating disease threatening the citrus industry worldwide. This disease is caused by a Gram-negative bacterium *Xanthomonas citri* subsp.*citri* (*Xcc*) [1, 2]. Research into citrus canker has been focused on the pathogen’s genome, host-pathogen interactions, resistant or susceptible genes of host. For example, Da Silva et al. [3] sequenced the genome of *Xcc-A306* strain, and found a large number of genes coding for cell wall degrading enzymes (*CWDEs*), proteases, type 2 secretion system (*T2SS*) and type 3 secretion system (*T3SS*). Whereas Zou et al. [4] identified the susceptibility gene *CsLOB1* in *Citrus* plants.

Plants produce a large number of specialized metabolites to defend themselves from environmental stresses such as attack from herbivores and pathogens [5–7]. The largest sector of these metabolites are the terpenoids, which are composed of isoprenoid units with highly diverse structures [8]. Thousands of terpenoid structures have been described. Based on the number of isoprenoid units in the molecules, terpenoids are classified as hemiterpenes (C5), monoterpenes (C10), sesquiterpenes (C15), diterpenes (C20), triterpenes (C30), tetraterpenes (C40), and their derivatives [9–11]. Hemi-, mono-, sesqui- and a few di-terpenes can be emitted to headspace as volatiles from particular organs, such as flowers, or after environmental stimulation such as oviposition, herbivory or wounding [12–14]. Di-, tri-, and tetraterpenes are mostly non-volatile compounds which increase in organs in response to (a)biotic stresses [15–17].

Volatile terpenoids are usually synthesized in plants through two pathways: the 2-C-methyl-D-erythritol 4-phosphate/1-deoxy-D-xylulose 5-phosphate pathway in plastids and the mevalonate pathway in the cytosol and peroxisomes [18, 19]. Terpene synthases (TPS) play important roles in these pathways by converting the precursor, geranyl diphosphate, into monoterpenes, farnesyl diphosphate into sesquiterpenes and geranylgeranyl diphosphate into diterpenes. *TPS* genes are found throughout the plant kingdom, from mosses to flower plants, and usually share structures and comprise medium-sized gene family, commonly divided into seven major branches from TPS-a to TPS-g by phylogenetic analysis[20]. For example, 40 *TPS*s were identified in the *Arabidopsis thaliana* genome [20], 27 in *Cucumis sativa* [21], and 152 in *Vitis vinifera* [22]. In *Citrus*, Dornelas et al. [23] found 49 *TPS*s by searching CitEST database. Whereas from genome sequences, 95 *TPS*s were identified in *Citrus sinensis* [24] and 58 in Finger citron (*C. medica var. sarcodactylis*) [25]. Like other terpenoids that respond to biotic stress, many *TPSs* are upregulated by herbivory [26–31].

Many terpenoids and *TPS*s are involved in the interactions between plants and their environment. For example, oviposition and injury by herbivores frequently elicits the release of terpenoid volatiles [13, 14, 16]. Light is also known to regulate terpenoids releases in several species [32]. Promoter regions of TPS genes have been analyzed and many stress-related *cis*-acting regulatory elements such as G-box elements, W-boxes elements, ABRE motifs and MYB binding sites are commonly found. The G-box elements are required for JA-mediated transcription regulation, which may be involved in herbivory responses, as well as being essential for light regulation [33]. W-boxes are associated with SA responses and ABRE motifs are associated with ABA responses [32]. W-boxes and MYB binding sites can specifically bind WRKY and MYB transcription factors that play important roles in disease resistance [34, 35].

Linalool is a monoterpene alcohol found in many plant species and has many different functions. It is a common component of floral scents [36], known to attract pollinators. In some cases, linalool and its derivatives can also act as an insect repellent [37, 38]. Linalool is also a volatile emitted from leaves. For example, in tomato, it mainly accumulates in trichomes [39]. Linalool is also found in insects where it may function as a pheromone [40] and a pathogen defense compound [41]. It is also in many natural essential oils, valued for its antibacterial activity against many gram-negative bacteria, with promise as a medicinal therapeutic [42]. In summary, linalool is widely found in the biological world with different context-dependent functions in interacting systems [43].

Citrus plants are often richly endowed in volatiles, among which linalool, limonene, caryophyllene and other terpenes are dominant components [44]. Shimada et al. [45] isolated and identified cDNAs of several monoterpene synthase genes including three linalool synthases from *C. unshiu*. Over-expression of citrus linalool synthase gene in sweet orange, which is susceptible to canker disease, enhanced its resistance to the pathogen [46]. Furthermore, linalool treatment inhibits the growth of *Xcc in vitro* [45], suggesting that linalool may function in citrus defense against *Xcc*. Given the context-dependence of linalool function, it is surprising that little is known about the transcriptional regulation of linalool synthase genes.

Here, we found that leaf linalool content varies greatly amongst 6 *Citrus* species and analyzed the promotor sequences both *in silico* and in an Arabidopsis reporter system, of two linalool synthase genes (*STS4*) from two *Citrus* species that divergently accumulate linalool and differ in *Xcc* resistance: *C. reticulata* and *C. grandis*.

## Results

### *C. reticulata* has higher leaf linalool contents than *C. grandis* and*C. medica*, and stronger *Xcc* resistance

**Fig. 1:**
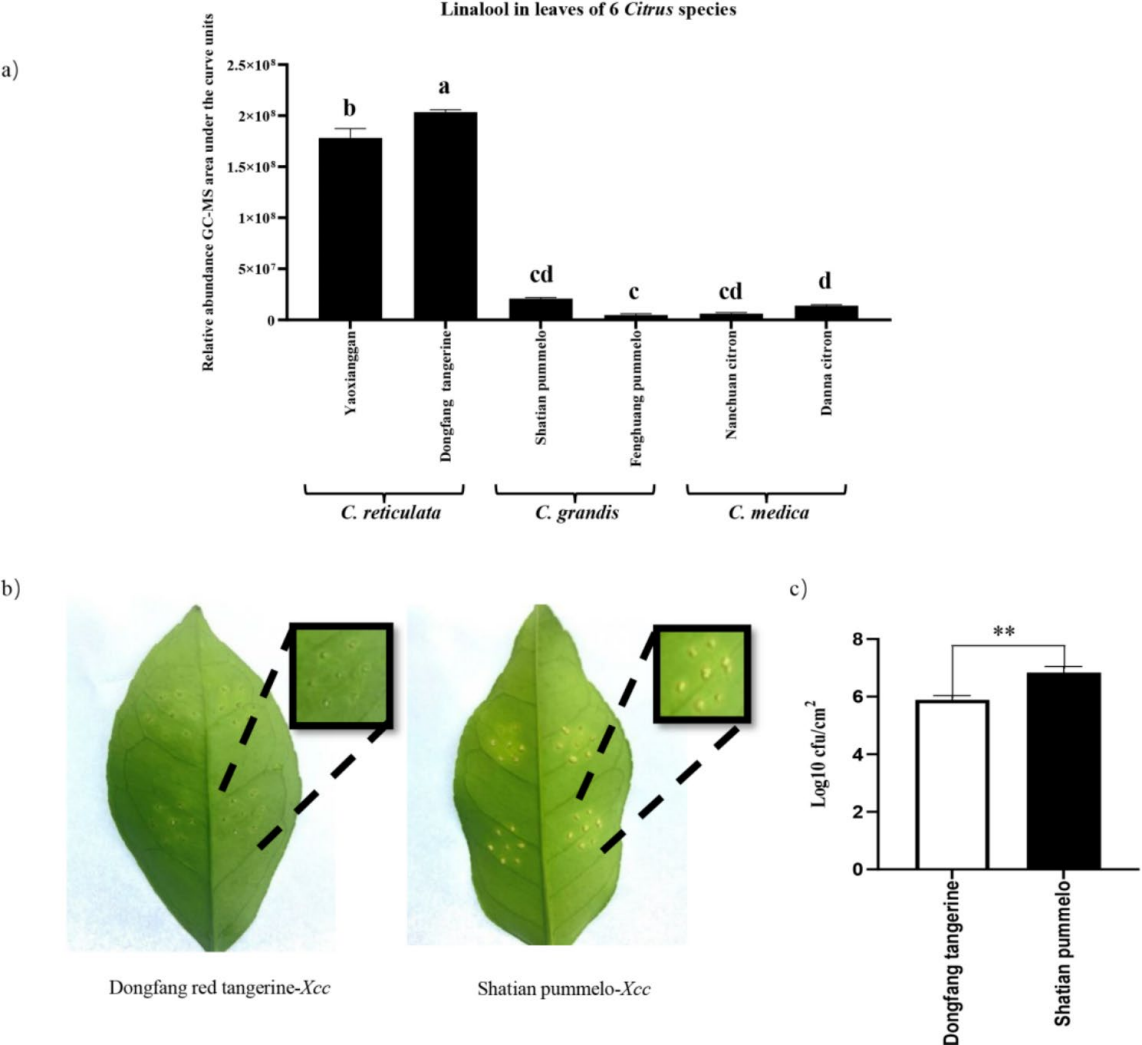
Variation of linalool in *Citrus* species and resistance against canker disease. **(a)** Relative linalool abundance in leaves of six *Citrus* cultivars including two *C. reticulata*, two *C. grandis* and two *C. medica*. The linalool content is much higher in two *C. reticulata* cultivars than in cultivars of the other two specie (n = 3, *p* < 0.05, ANOVA) s. **(b)** Leaves of a *C. reticulata* (“Dongfang” tangerine) and a *C. grandis* (“Shatian” pummelo) infected by *Xanthomonas citri subsp.citri*. Shown are representative leaves from experiments repeated three times, with at least three replicates in each treatment group. **(c)** Colony forming units (cfu) were counted at seven sites to assess the accumulation of bacterial populations (n = 3, * * *p* < 0.01, *t*-test)

To investigate the variation of foliar linalool contents, six *Citrus* accessions including 2 *C. grandis*, 2 *C. medica* and 2 *C. reticulata* were selected to measure the internal linalool content in their leaves. We found that linalool contents in *C. reticulata leaves* were much higher than that in *C. grandis* and *C. medica* leaves, about 8–42 fold higher (Fig. 1a).

Previous studies showed that linalool in citrus plants may mediate resistance against *Xanthomonas citri* subsp.*citri* (*Xcc*). We infested “Dongfang” tangerine (high linalool) and “Shatian” pummelo (low linalool) with *Xcc*. Ten days after infestation of the pathogen, “Dongfang” tangerine showed smaller sponge lesions and fewer colonies (Fig. 1b 1c) than did “Shatian” pummelo, indicating stronger resistance to *Xcc*.

### A linalool synthase gene is only highly transcribed in *C. reticulata*

**Fig. 2:**
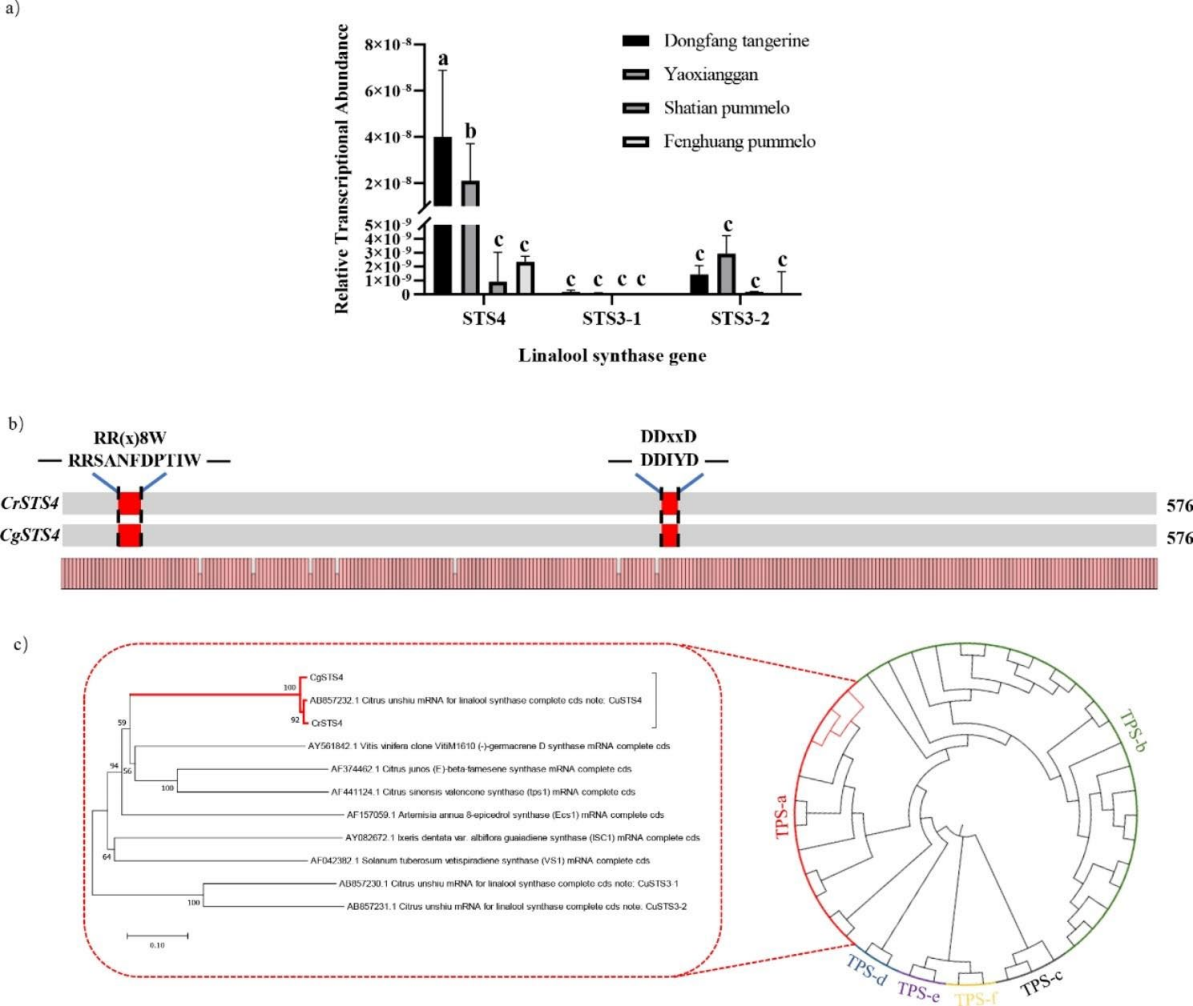
Variation in transcript levels of linalool synthase genes and in coding sequences of the major leaf-expressed *STS4*in citrus species. **(a)** Relative transcript abundances of three linalool synthase genes in leaves of different citrus cultivars (n = 3, *p* < 0.05, ANOVA). **(b)** Alignment of amino acid sequences encoded by the orthologs of the major leaf-expressed *STS4* between *C. reticulata* (*CrSTS4*) and *C. grandis* (*CgSTS4*). Typical terpene synthase motifs are indicated in red. **(c)** Phylogenetic relationship between linalool synthase gene and plant terpene synthases (TPSs) in *CrSTS4* and *CgSTS4*. The tree was constructed using the neighbor-joining (NJ) method

Shimada et al. [45] identified three linalool synthase genes *STS4*, *STS3-1* and *STS3-2* in citrus. In order to study which genes may control linalool levels in leaves, we performed qPCR analysis of these three genes in six varieties (Fig. 2a, Fig. S1), finding that *STS4* show the highest expression level in leaves. Combined with linalool measurement results shown previously, we selected the representative genotypes “Dongfang” tangerine and “Shatian” pummelo for subsequent research into the role of *STS4* in resistance to *Xcc*.

We isolated the cDNA of this gene from leaves of “Dongfang” tangerine and “Shatian” pummelo (*CrSTS4* and *CgSTS4*, respectively). The amino acid sequence alignment of *CrSTS4* and *CgSTS4* showed that they were highly similar (98.8%). The typical motifs for active mono-*TPS* genes such as RR(x)8 W and DDxxD are present in both genes (Fig. 2b). A phylogenic analysis of the 2 *STS4* genes among previously characterized TPSs revealed that both genes were in the TPS-a subfamily, and the closest gene was the linalool synthase gene *CuSTS4* identified in *C. unshiu* (Fig. 2c).

### *STS4* promoter regions differ between *C. reticulata* and *C. grandis* and harbor multiple stress-related *cis*-acting elements

**Fig. 3:**
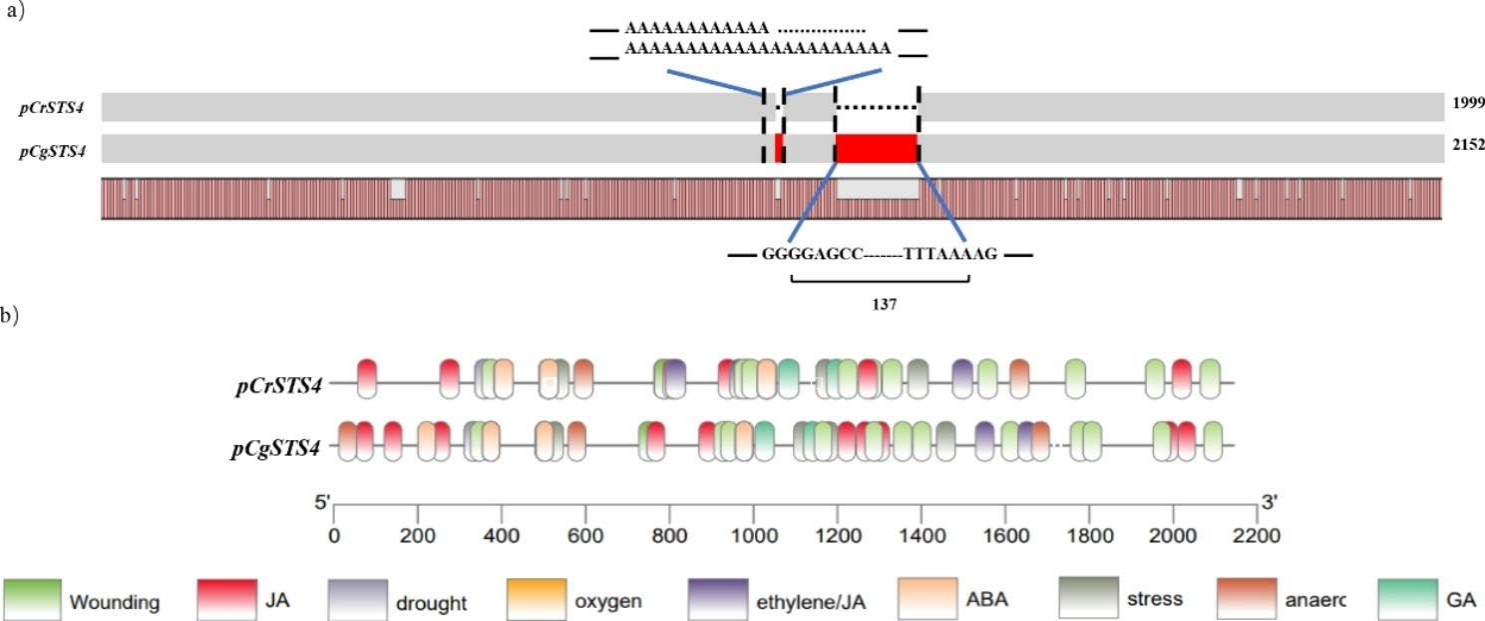
Analysis of *STS4* promoter sequences in *C. reticulata* and *C. grandis*. **(a)** Nucleic acid sequence alignment of *pCrSTS4* (promoter of *STS4* isolated from *C. reticulata*) and *pCgSTS4* (promoter of *STS4* isolated from *C. grandis*). The different areas are in red. **(b)** Analysis of putative *cis*-acting elements in *CrSTS4* and *CgSTS4*. Putative functions of the motifs are indicated by different colors

To reveal the mechanism responsible for the different accumulations of *CrSTS4* and *CgSTS4* transcripts, we sequenced 1999 and 2152 bp upstream of the start codons of *CrSTS4* and *CgSTS4*, respectively. Alignment of these promoter sequences revealed an identity of 93.89% (Fig. 3a), and a number of SNPs and insertion/deletion variations between them. Remarkably, a 137 bp sequence was only present in the promoter region of *C. grandis.*

We analyzed the potential *cis*-regulatory elements of the two promoters using PlantCARE (Fig. 3b, Table S1). The *CrSTS4* and *CgSTS4* promoters shared most of the elements which might be regulated by environmental factors such as light, hormones and mechanical damage. The *CgSTS4* promoter has an AAGAA-motif element and a GATA-motif element lacking in the *CrSTS4* promoter, in addition to MYB and MYC elements, implying that the *CgSTS4* gene may be more strongly regulated by environmental stresses.

### In transgenic Arabidopsis, the *CrSTS4* promoter showed stronger constitutive activity in trichomes

**Fig. 4:**
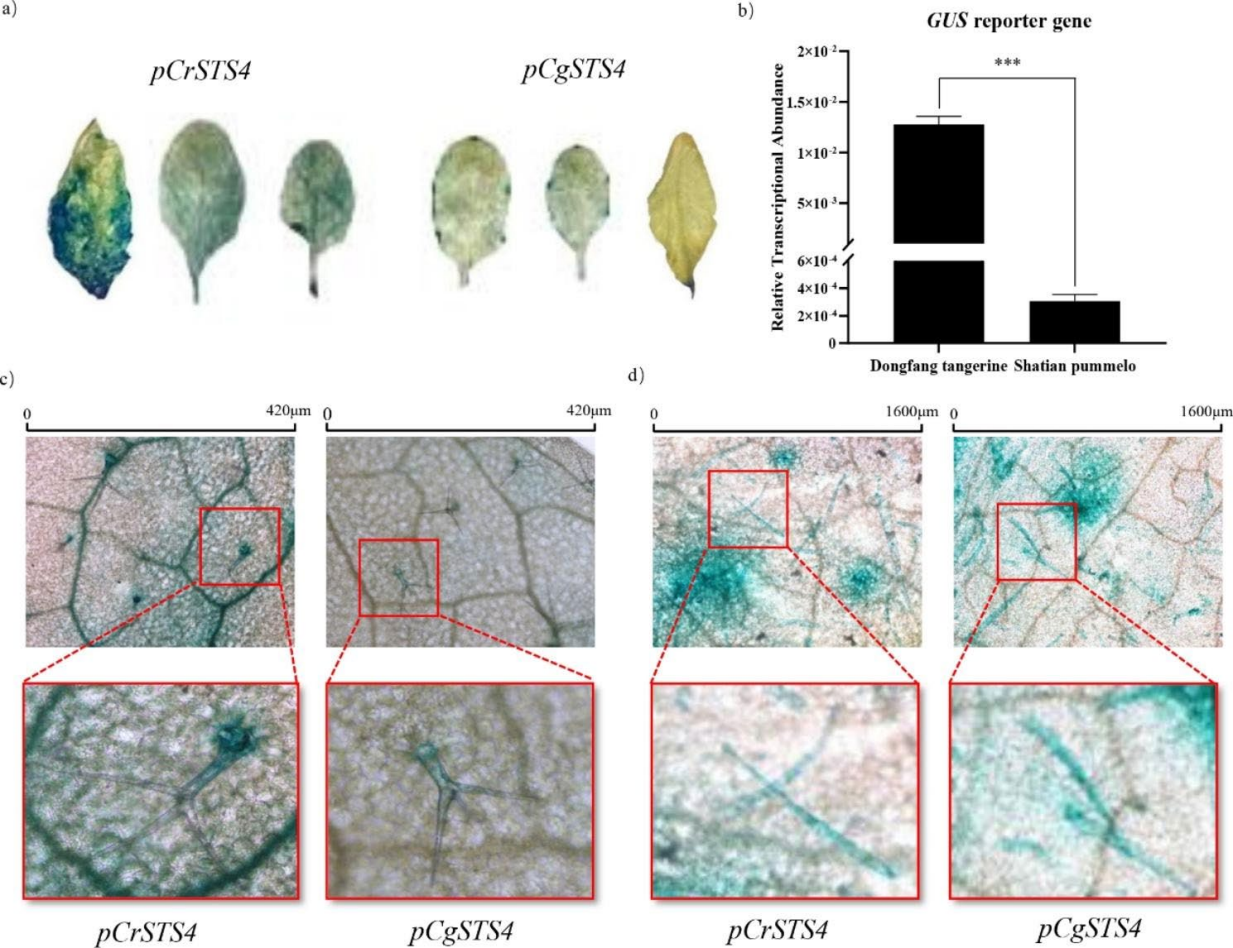
*pCrSTS4* exhibits stronger constitutive activity than *pCgSTS4* after transformation into Arabidopsis, specifically in veins and trichomes. **(a)***GUS* staining analysis of the third generation homozygous transgenic *Arabidopsis thaliana* harboring *pCrSTS4::GUS* or *pCgSTS4::GUS* (n = 3, * * *p < 0.001, *t*-test). **(b)** The transcript abundance of the *GUS* gene in transgenic Arabidopsis plants. **(c)** Both *pCrSTS4* and *pCgSTS4* were active in the trichomes in the transgenic Arabidopsis, *pCrSTS4* was also active in the veins. **(d)** GUS-stained tobacco leaves transiently expressing *pCrSTS4* and *pCgSTS4.* Similar expression patterns were present for both promoters. c) and d) were observed with an optical microscope

In order to reveal the activity of the promoters of *CrSTS4* (*pCrSTS4*) and *CgSTS4* (*pCgSTS4*), we fused them with a *GUS* reporter gene and transformed them into *Arabidopsis*. GUS-staining of the rosette leaves revealed that the *GUS* reporter driven by *pCrSTS4* was much more strongly expressed than that driven by *pCgSTS4* (Fig. 4a 4b).

In order to reveal the spatial expression of *CrSTS4* and *CgSTS4*, the GUS-stained Arabidopsis leaves were observed under an optical microscope. In both *pCrSTS4::GUS* and *pCgSTS4::GUS* transgenic plants, the trichomes were strongly stained. Whereas veins and mesophyll cells were also stained only in *pCrSTS4::GUS* plants (Fig. 4c).

We further assessed spatial expression by transiently expression of *pCrSTS4::GUS* and *pCgSTS4::GUS* constructs in cultivated tobacco leaves using *Agrobacterium* infiltration. Staining of the infiltrated leaves revealed a similar spatial expression as in Arabidopsis, being strong in the trichomes (Fig. 4d).

### *Pst DC3000* and phytohormone elicitation of *CrSTS4* and *CgSTS4* expression in transgenic Arabidopsis

**Fig. 5:**
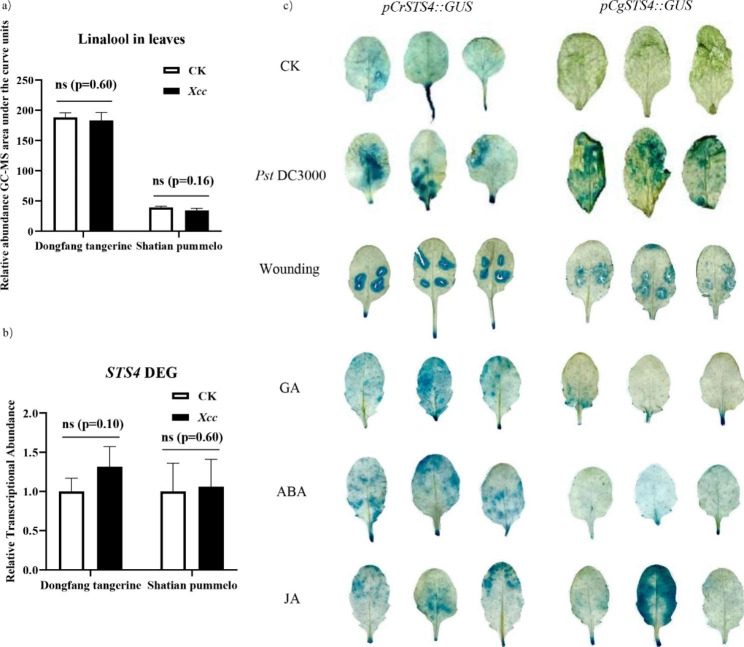
Linalool and transcription of STS4 in citrus do not response to *Xcc* infection but *pCrSTS4* and *pCgSTS4* are activated by infection with bacterial pathogen, wounding and phytohormones in transgenic Arabidopsis. **(a)** Relative abundance of linalool in leaves of *C. reticulata* and *C. grandis* plants with or without *Xcc* infection (n = 3, *t*-test). **(b)** Transcript levels of *CrSTS4* and *CgSTS4* in leaves of infected or noninfected citrus plants, measured with qRT-PCR (n = 3, *t*-test). **(c)** GUS-stained leaves of transgenic Arabidopsis plants harboring *pCrSTS4* or *pCgSTS4* challenged by *Pst* DC3000, mechanical damage, and treatment of external GA, ABA and JA

Many terpenoids are known to be induced by biological stresses such as herbivory or pathogen attack. We tested if linalool in “Dongfang” tangerine and “Shatian” pummelo was inducible upon infestation of *Xcc.* Three days after *Xcc* inoculation, both tangerine and pummelo showed no difference in linalool contents compared to the controls (Fig. 5a). Similar results were found with Mangshanyeju (*C. reticulata*) and kumquat (*Poncirus*) (Fig. S2a). Consistently, the transcript abundance of the *STS4* gene in these varieties was also not influenced by the elicitations (Fig. 5b, Fig. S2b). However, since we found a number of stress related *cis*-acting elements present in their promoter regions, we tested the transgenic Arabidopsis plants for their responses to the bacterial pathogen *Pst* DC3000. Interestingly, GUS-staining revealed that both *pCrSTS4* and *pCgSTS4* were strongly activated (Fig. 5c). We further found that the activities of the two promoters were also induced by mechanic wounding and exogenous treatment with JA. However, only *pCrSTS4* responded to gibberellin (GA3) and abscisic acid (ABA) treatments (Fig. 5c).

## Discussion

Linalool is present in more than 200 monocotyledonous and dicotyledonous plants. In particular, many plants in Labiatae, Lauraceae and Rutaceae contain large amounts of linalool. We found considerable variation in foliar linalool contents in different *Citrus* species. *Citrus reticulata*, such as “Shatang” and “Dongfang” tangerine contains more linalool than *C. grandis* accessions such as “Fenghuang” and “Shatian” pummelo and *C. medica* accessions such “Nanchuan” and “Danna” citron. Linalool is an important defensive metabolite. As a volatile cue, it can directly repel some insects [37], and mediate tri-trophic interactions between plants, herbivores and natural enemies [38, 43]. Moreover, linalool has broad-spectrum resistance to a variety of pathogenic microorganisms [47], including a variety of human oral bacterial pathogens [48] and the pathogenic fungus *Alternaria alternata* [49]. Droby et al. [50] reported that linalool inhibits the germination of *Penicillium italicum* and *P. digitatum* spores. And linalool was found to have the antibacterial activity against *Acinetobacter baumanni* [51]. In citrus, linalool inhibits the growth of *Xcc* and *P. italicum*, the pathogen of postharvest rot disease of citrus [45]. Furthermore, in different citrus varieties the content of linalool appears to be associated with resistance to *Xcc* [52]. Overexpression of a linalool synthase gene in the citrus variety “Hamlin” sweet orange increased resistance to canker disease [46]. In our study, “Dongfang” tangerine, a variety with high linalool abundance, showed stronger resistance to *Xcc* than “Shatian” pummelo, both in lesion size and colony statistics. A previous study which compared long-term canker-resistance among 186 citrus genotypes in the field found that many tangerine (*C. reticulata*) genotypes were among the most resistant ones [53]. On the other hand, even though the *C. grandis* genotypes were not included in this study, its close relative, *C. paradise* are among the most susceptible genotypes of this study. This difference in resistance may be due to many factors including cuticle thickness of the leaf, wax content, stomata density and linalool content, as revealed in this study.

Terpene synthase (TPS) is responsible for the synthesis of various terpene molecules from precursors. Intra-specific variation of terpenoids could be caused by the variation in responsible terpene synthase in different varieties of same species or closely related species. For example, we previously found that in different varieties of the wild tobacco, *Nicotiana attenuata*, a linalool synthase has two alleles. One allele encodes an enzyme with full efficiency to synthesize linalool, while the other allele harbors a deletion in the coding sequence and is not correctly spliced, to encode an enzyme inefficient in synthesizing linalool. This allelic variation accounts the differences in linalool accumulation among geographically interspersed conspecific wild tobacco plants [38]. However, in this study, variation in linalool among *Citrus*. spp seems to be controlled by the different *STS4* transcript abundances levels. This inference is based on the observations that the isolated CDS of *CrSTS4* and *CgSTS4* harbored only minor variations and both encoded enzymes with fully functional TPS domains. Moreover, *CrSTS4* transcript levels in tangerine are much higher than those of *CgSTS4* in pummelo, which is consistent with their linalool contents. We found larger differences in the promoter regions than that in coding sequence of *STS4* between the two *Citrus* species. Although we can not exclude the possibility that the minor variation in coding sequences of *CrSTS4* and *CgSTS4* could account for differences in the rates of linalool biosynthesis, the available data is consistent with transcriptional regulation of linalool. This inference is also consistent with the observation that *pCrSTS4* exhibited stronger activity than *pCgSTS4* when transformed into Arabidopsis.

Both *pCrSTS4* and *pCgSTS4* are specifically active in trichomes, both in Arabidopsis and in tobacco. The trichomes are the first line of defense against stress in plants, and trichomes can directly sense external mechanical forces to predict pathogen infection [54]. However, there are no trichome structure on citrus leaves, which instead have thick oil glands that resist stress and pathogen invasion [55]. Some TPS genes have been found to be specifically transcribed in the epithelial cells surrounding the oil glands in rough lemon leaf [56] and it is likely that *STS4* is also expressed in a similar tissue. However, additional experiments in citrus are required to verify this hypothesis.

Many terpenoids and their synthase genes are responsive to environmental stresses such as herbivores, pathogens, and mechanical damage or external methyl jasmonate(MeJA. This kind of induction has been reported in a number of plant species including conifers [57, 58], tomato [28, 29], maize [30], leguminous plants [26] and cucumber [32]. In our study the variation of linalool and transcription of *STS4* are constitutive present in the *Citrus* spp. Previous study showed that *STS4* in *C. unshiu* was upregulated by infestation of *Xcc* [45]. However, later study from the same laboratory reported conflicting results [52]. We found a number of stress-related or hormone-responsive *cis*-acting regulatory elements present in the promoters of *STS4* isolated from two citrus species (*pCrSTS4* and *pCgSTS4*), but did not find significant alternations in linalool abundance in both tangerine and pummelo after infestation of *Xcc*. Consistently, transcript levels of *STS4* in the two species were also not induced by *Xcc*. Interestingly, *pCrSTS4* and *pCgSTS4* transferred into Arabidopsis were significantly activated by infection of a bacterial pathogen *Pst* DC3000. This could be because *STS4* is a defense-related gene and is generally responsive to invading pathogens. However, this general ability could be inactivated in a specific susceptible interaction between *Citrus* plants and *Xcc.* Furthermore, we found that *pCrSTS4* and *pCgSTS4* were also activated by mechanical damage and external JA, implying that *STS4* might also play a role in defense against other stresses such as herbivory, responses which are commonly regulated by the JA signaling pathway.

## Conclusions

In conclusion, this study found that linalool content in tangerine leaves is higher than in other citrus species and has stronger resistance to *Xcc*. This high level of linalool is associated with higher transcript levels of a linalool synthase gene. The promoter of this gene from tangerine shows stronger activities than that from pummelo, after being transformed into Arabidopsis. Interestingly, although this gene is not induced by *Xcc* infection, its promoter is activated by *Pst* DC3000 in transgenic *Arabidopsis thaliana.* This study provides insights into how constitutive and induced terpene synthase genes combat bacterial pathogens in *Citrus*, information which could be useful for breeding resistant varieties of *Citrus*.

## Materials and methods

### Plant material and pathogen inoculation

“Dongfang” tangerine (*C. reticulata*), “Shatian” pummelo (*C. grandis*) and other plant samples used in this study were from the National Citrus Germplasm Repository in Chongqing, China. *Xcc* was from a culture maintained in our laboratory. The leaves were surface-sterilized with 75% ethanol on a sterile bench before inoculation with *Xcc*. A wound in the leaf lamina was created using a needle (0.5 mm). The double distilled water re-suspended *Xcc* suspension (OD_600_ = 0.6) was injected to about 3/4 of the leaf area; water was injected as a control. After inoculation, leaves were cultured on a sterile petri dish and the petioles were wetted. After 3 days of culture in the incubator (28℃, 60% humidity), the injected area of leaf was taken for subsequent experiments. Each experiment included three biological replicates.

Using the same materials, wounds were created with needles (0.5 mm), and inoculated with 1 µl of each *Xcc* suspension (OD_600_ = 0.6). Ulcer symptoms were imaged at 10 days after inoculation. Three lesions were thoroughly ground in double distilled water. After continuous dilution, the liquid was spread on an LB solid plate and cultured at 28 °C for 3 days. The colony forming units of the three lesions were counted.

The *Pst* DC3000 pathogen challenge was performed with the transgenic Arabidopsis harboring promoter regions of *CrSTS4* and *CgSTS4* following the method of Fang et al. [59] with minor modifications. *Pst* DC3000 was cultured in KB medium containing 50 mg/ml rifampicin to OD_600_ = 0.5. The culture was centrifuged at 5000 g for 5 min, and resuspended in sterile 10 mM MgCl_2_ buffer to OD_600_ = 0.0005. Then, the bacteria were inoculated into the adaxial side of 4-week-old homozygous transgenic *Arabidopsis thaliana* leaves using a needle-free 1 mL syringe, and 10 mM MgCl_2_ buffer was injected as a control. Each experiment was repeated five times.

### GC-MS analysis

One hundred mg of *Xcc* treated citrus leaves were ground in liquid nitrogen, re-suspended in 5 mL saturated NaCl solution in a glass vial with a stir bar. Cyclohexanone was added to the solution as the internal standard. The vial was sealed with a septum screw-top cap. The samples were equilibrated in a water bath at 40 °C for 20 min, and volatile compounds were collected by solid phase microextraction (SPME) method. A fiber coated with divinylbenzene/carboxen/polydimethylsiloxane (DVB/CAR/PDMS, Supelco, Bellefonte, PA) was exposed at the top space of the capped vial for 30 min. The SPME fibers were desorbed for 5 min. The volatiles were determined using an Agilent 7890B gas chromatography and an Agilent 5977 A mass spectrometry. Volatile compounds were identified by comparing its retention time and mass spectrometry matching the mass spectral library (NIST11, W10N14).

### Cloning and qRT-PCR analysis of linalool synthase gene

The sequences of linalool synthase genes in tangerine (*CrSTS4*) and pummelo (*CgSTS4*) genomes were retrieved using BLAST in Citrus Pan-genome to Breeding Database (http://citrus.hzau.edu.cn/download.php). Biospin Plant Total RNA Extraction Kit (BioFlux) was used to extract total RNA from the leaves of “Dongfang” tangerine and “Shatian” pummelo inoculated with *Xcc* and water controls. cDNA was synthesized and used as template (primers in Table S2) to amplify the linalool synthase genes from the two varieties using the PrimeSTAR Max DNA polymerase (TaKaRa). The obtained PCR product was purified and connected using the 5minTM TA / Blunt-Zero Cloning Kit (Vazyme), and transformed into *E.coli* Mach1-T1 competent cells for culture and sequencing. The comparison analysis was performed using CLC Sequence Viewer 7.

The cDNA was used as a template, qPCR primers (Table S2) were designed, and *CitActin* was used as internal reference gene. The cycle procedure was as follows: initial denaturation at 95 °C for 30 s, and then amplification for 40 cycles (95 °C 10 s, 58 °C 30 s, 72 °C 30 s). By associating the Ct value of the expression level with the Ct value of the reference gene *CitActin*, the cycle threshold (Ct) value of the original data was converted to a standardized expression level by the 2^−ΔCt^ method (27).

### Cloning of promoter region and generation of transgenic arabidopsis

In Citrus Pan-genome to Breeding Database (http://citrus.hzau.edu.cn/index.php), the 1999 bp (*C. reticulata* v1.0) and 2152 bp (*C. grandis* (L.) Osbeck.cv. “ Wanbaiyou” v1.0) fragments upstream of the start codon of *CrSTS4* (*pCrSTS4*) and *CgSTS4* (*pCgSTS4*) were extracted from the genomic sequences for primer design, respectively (Table S2).

By ClonExpress II One Step Cloning Kit (Vazyme) kit, *pCrSTS4 and pCgSTS4* were ligated to the pCambia-2016-GUS vector, respectively. A *promoter::GUS* reporter gene system was constructed and transformed into the *E. coli* clone strain Mach1-T1 and then into *Agrobacterium tumefaciens GV3101* strain. Transformation of Arabidopsis was performed by flower infiltration [60]. The seeds were screened for positive constructs using MS-Kanamycin (50 mg / mL) solid medium.

### Promoter *cis* -acting element analysis

The PlantCARE database (http://bioinformatics.psb.ugent.be/webtools/plantcare/) was used to analyze the *cis*-acting elements present in *pCrSTS4 and pCgSTS4*. The alignment motifs of each promoter are listed as their distance from the start codon of the gene.

### Activity identification of promoter region and optical microscope observation

Four-week-old T_3_ homozygous transgenic *Arabidopsis thaliana* reporter plants were screened. During the active photoperiod, leaves of similar size in the same part of the plants were taken and stained with GUS Stain Kit (Solarbio) at 37 °C for 8 h. The solution discoloration and blue spots on the leaves could be observed by the naked eyes. After 70% alcohol decolorization, leaves were photographed, and placed on a glass slide for examination with an optical microscope.

### Elicitation experiments of transgenic arabidopsis

Following the methods of He et al. [32], leaves of the transgenic Arabidopsis plants were challenged by: mechanical damage (using a needle with a diameter of 0.2 mm to puncture the blade); hormone treatments with jasmonic acid(JA), abscisic acid(ABA), and gibberellin (GA3) (all in 5 µl of 1 mM + 0.01% Tween-20), were followed by GUS Stain Kit (Solarbio) staining at 37 °C for 8 h, and 70% alcohol fading for observation and photographing.

### Electronic supplementary material

Below is the link to the electronic supplementary material.

Supplementary Material 1

## Data Availability

The datasets used and/or analyzed during the current study, are available from the corresponding author on reasonable request.

## Abbreviations

*Xcc*: *Xanthomonas citri* subsp. *Citri*
*Pst* DC3000: *Pseudomonas syringae* DC3000
JA: Jasmonic acid
ABA: Abscisic acid
GA3: Gibberellin
MeJA: Methyl jasmonate
TPS: Terpene synthase

## References

[1] Omar AA, Murata MM, El-Shamy HA, Graham JH, Grosser JW (2018). Enhanced resistance to citrus canker in transgenic mandarin expressing *Xa21* from rice. Transgenic Res.

[2] Schaad NW, Postnikova E, Lacy GH, Sechler A, Agarkova I, Stromberg PE, Stromberg VK, Vidaver AK (2005). Reclassification of Xanthomonas campestris pv. Citri (ex Hasse 1915) dye 1978 forms a, B/C/D, and E as X. smithii subsp citri (ex Hasse) sp nov nom. rev. comb. nov., X. fuscans subsp aurantifolii (ex Gabriel 1989) sp nov nom. rev. comb. nov., and X. alfalfae subsp citrumelo (ex Riker and Jones) Gabriel et al., 1989 sp nov nom. rev. comb. nov.; X. campestris pv malvacearum (ex Smith 1901) dye 1978 as X. smithii subsp smithii nov comb. nov nom. nov.; X. campestris pv. Alfalfae (ex Riker and Jones, 1935) dye 1978 as X. alfalfae subsp alfalfae (ex Riker et al., 1935) sp nov nom. rev.; and “var. fuscans” of X. campestris pv. Phaseoli (ex Smith, 1987) dye 1978 as X. fuscans subsp fuscans sp nov. Syst Appl Microbiol.

[3] Da Silva AC, Ferro JA, Reinach FC, Farah CS, Furlan LR, Quaggio RB, Monteiro-Vitorello CB, Van Sluys MA, Almeida NF, Alves LM (2002). Comparison of the genomes of two *Xanthomonas* pathogens with differing host specificities. Nature.

[4] Zou XP, Du MX, Liu YN, Wu L, Xu LZ, Long Q, Peng AH, He YR, Andrade M (2021). Chen SC *CsLOB1* regulates susceptibility to citrus canker through promoting cell proliferation in citrus. Plant J.

[5] Huang AC, Jiang T, Liu YX, Bai YC, Reed J, Qu B, Goossens A, Nutzmann HW, Bai Y (2019). Osbourn A A specialized metabolic network selectively modulates *Arabidopsis* root microbiota. Science.

[6] Kessler A, Baldwin IT (2001). Defensive function of herbivore-induced plant volatile emissions in nature. Science.

[7] Lee C, Kim T, Seung N-PR (2014). Rhee genomic signatures of specialized metabolism in plants. Science.

[8] Aharoni A, Jongsma MA, Bouwmeester HJ (2005). Volatile science? Metabolic engineering of terpenoids in plants. Trends Plant Sci.

[9] Sawamura M (2000). Volatile components of essential oils of the *Citrus* genus. Recent Resdevelagricultural & Food Chem.

[10] Vekiari SA, Protopapadakis EE, Papadopoulou P, Papanicolaou D, Panou C, Vamvakias M (2002). Composition and seasonal variation of the essential oil from leaves and peel of a cretan lemon variety. J Agric Food Chem.

[11] Zhang M, Su P, Zhou YJ, Wang XJ, Zhao YJ, Liu YJ, Tong YR, Hu TY, Huang LQ, Gao W (2015). Identification of geranylgeranyl diphosphate synthase genes from *Tripterygium wilfordii*. Plant Cell Rep.

[12] Cao R, Zhang Y, Mann FM, Huang C, Mukkamala D, Hudock MP, Mead ME, Prisic S, Wang K, Lin FY (2010). Diterpene cyclases and the nature of the isoprene fold. Proteins.

[13] Herde M, Gartner K, Kollner TG, Fode B, Boland W, Gershenzon J, Gatz C, Tholl D (2008). Identification and regulation of TPS04/GES, an Arabidopsis geranyllinalool synthase catalyzing the first step in the formation of the insect-induced volatile C16-homoterpene TMTT. Plant cell.

[14] Hilker M, Fatouros NE (2015). Plant responses to insect egg deposition. Annu Rev Entomol.

[15] Balkema-Boomstra AG, Zijlstra S, Verstappen FW, Inggamer H, Mercke PE, Jongsma MA, Bouwmeester HJ (2003). Role of cucurbitacin C in resistance to spider mite (*Tetranychus urticae*) in cucumber (*Cucumis sativus* L). J Chem Ecol.

[16] Bohlmann J, Martin D, Oldham NJ, Gershenzon J (2000). Terpenoid secondary metabolism in *Arabidopsis thaliana*: cDNA cloning, characterization, and functional expression of a myrcene/(E)-beta-ocimene synthase. Arch Biochem Biophys.

[17] Nagegowda DA (2010). Plant volatile terpenoid metabolism: biosynthetic genes, transcriptional regulation and subcellular compartmentation. FEBS Lett.

[18] Lichtenthaler HK (1999). The 1-deoxy-D-xylulose-5-phosphate pathway of isoprenoid biosynthesis in plants. Ann Rev Plant Physiol Plant Mol Biol.

[19] Sapir-Mir M, Mett A, Belausov E, Tal-Meshulam S, Frydman A, Gidoni D, Eyal Y (2008). Peroxisomal localization of *Arabidopsis* isopentenyl diphosphate isomerases suggests that part of the plant isoprenoid mevalonic acid pathway is compartmentalized to peroxisomes. Plant Physiol.

[20] Chen F, Tholl D, Bohlmann J, Pichersky E (2011). The family of terpene synthases in plants: a mid-size family of genes for specialized metabolism that is highly diversified throughout the kingdom. Plant J.

[21] He J, Bouwmeester HJ, Dicke M, Kappers IF (2020). Genome-wide analysis reveals transcription factors regulated by spider-mite feeding in Cucumber (*Cucumis sativus*). Plants (Basel).

[22] Martin DM, Aubourg S, Schouwey MB, Daviet L, Schalk M, Toub O, Lund ST, Bohlmann J (2010). Functional annotation, genome organization and phylogeny of the grapevine (*Vitis vinifera*) terpene synthase gene family based on genome assembly, FLcDNA cloning, and enzyme assays. BMC Plant Biol.

[23] Dornelas MC (2007). Mazzafera P A genomic approach to characterization of the *Citrus* terpene synthase gene family. Genet Mol Biol.

[24] Alquezar B, Rodriguez A, de la Pena M, Pena L (2017). Genomic analysis of terpene synthase family and functional characterization of seven sesquiterpene synthases from *Citrus sinensis*. Front Plant Sci.

[25] Xu Y, Zhu C, Xu C, Sun J, Grierson D, Zhang B, Chen K (2019). Integration of metabolite profiling and transcriptome analysis reveals genes related to volatile terpenoid metabolism in Finger Citron (*C. medica* var. *sarcodactylis*). Molecules.

[26] Arimura G, Huber DP, Bohlmann J (2004). Forest tent caterpillars (*Malacosoma disstria*) induce local and systemic diurnal emissions of terpenoid volatiles in hybrid poplar (*Populus trichocarpa x deltoides*): cDNA cloning, functional characterization, and patterns of gene expression of (-)-germacrene D synthase, *PtdTPS1*. Plant J.

[27] De Vos M, Van Oosten VR, Van Poecke RM, Van Pelt JA, Pozo MJ, Mueller MJ, Buchala AJ, Metraux JP, Van Loon LC, Dicke M (2005). Signal signature and transcriptome changes of *Arabidopsis* during pathogen and insect attack. Mol Plant Microbe Interact.

[28] Kant MR, Ament K, Sabelis MW, Haring MA, Schuurink RC (2004). Differential timing of spider mite-induced direct and indirect defenses in tomato plants. Plant Physiol.

[29] Martel C, Zhurov V, Navarro M, Martinez M, Cazaux M, Auger P, Migeon A, Santamaria ME, Wybouw N, Diaz I (2015). Tomato whole genome transcriptional response to *Tetranychus urticae* identifies divergence of spider mite-induced responses between tomato and *Arabidopsis*. Mol Plant Microbe Interact.

[30] Schnee C, Kollner TG, Gershenzon J, Degenhardt J (2002). The maize gene terpene synthase 1 encodes a sesquiterpene synthase catalyzing the formation of (*E*)-beta-farnesene, (*E*)-nerolidol, and (*E*,*E*)-farnesol after herbivore damage. Plant Physiol.

[31] Zhurov V, Navarro M, Bruinsma KA, Arbona V, Santamaria ME, Cazaux M, Wybouw N, Osborne EJ, Ens C, Rioja C (2014). Reciprocal responses in the interaction between *Arabidopsis* and the cell-content-feeding chelicerate herbivore spider mite. Plant Physiol.

[32] He J, Verstappen F, Jiao A, Dicke M, Bouwmeester HJ (2022). Kappers IF terpene synthases in cucumber (*Cucumis sativus*) and their contribution to herbivore-induced volatile terpenoid emission. New Phytol.

[33] Vom Endt D, Soares e Silva M, Kijne JW, Pasquali G, Memelink J (2007). Identification of a bipartite jasmonate-responsive promoter element in the *Catharanthus roseus ORCA3* transcription factor gene that interacts specifically with AT-Hook DNA-binding proteins. Plant Physiol.

[34] Raffaele S, Rivas S (2013). Regulate and be regulated: integration of defense and other signals by the AtMYB30 transcription factor. Front Plant Sci.

[35] Xu XP, Chen CH, Fan BF, Chen ZX (2006). Physical and functional interactions between pathogen-induced *Arabidopsis* WRKY18, WRKY40, and WRKY60 transcription factors. Plant Cell.

[36] Knudsen JT, Eriksson R, Gershenzon J, Stahl B (2006). Diversity and distribution of floral scent. Bot Rev.

[37] Boachon B, Junker RR, Miesch L, Bassard JE, Hofer R, Caillieaudeaux R, Seidel DE, Lesot A, Heinrich C, Ginglinger JF (2015). CYP76C1 (cytochrome P450)-mediated linalool metabolism and the formation of volatile and soluble linalool oxides in *Arabidopsis* flowers: a strategy for defense against floral antagonists. Plant Cell.

[38] He J, Fandino RA, Halitschke R, Luck K, Kollner TG, Murdock MH, Ray R, Gase K, Knaden M, Baldwin IT (2019). An unbiased approach elucidates variation in (*S*)-(+)-linalool, a context-specific mediator of a tri-trophic interaction in wild tobacco. Proc Natl Acad Sci U S A.

[39] Van Schie CC, Haring MA (2007). Schuurink RC Tomato linalool synthase is induced in trichomes by jasmonic acid. Plant Mol Biol.

[40] Rudmann AA, Aldrich JR (1987). Chirality determinations for a tertiary alcohol - ratios of linalool enantiomers in insects and plants. J Chromatogr.

[41] Giglio A, Brandmayr P, Dalpozzo R, Sindona G, Tagarelli A, Talarico F, Brandmayr TZ (2009). Ferrero EA the defensive secretion of *Carabus lefebvrei* Dejean 1826 pupa (Coleoptera, Carabidae): gland ultrastructure and chemical identification. Microsc Res Tech.

[42] Maczka W, Duda-Madej A, Grabarczyk M, Winska K (2022). Natural compounds in the battle against microorganisms-linalool. Molecules.

[43] Raguso RA (2016). More lessons from linalool: insights gained from a ubiquitous floral volatile. Curr Opin Plant Biol.

[44] Zhang HP, Chen MJ, Wen H, Wang ZH, Chen JJ, Fang L, Zhang HY, Xie ZZ, Jiang D, Cheng YJ (2020). Transcriptomic and metabolomic analyses provide insight into the volatile compounds of citrus leaves and flowers. BMC Plant Biol.

[45] Shimada T, Endo T, Fujii H, Rodriguez A, Pena L, Omura M (2014). Characterization of three linalool synthase genes from Citrus unshiu Marc. And analysis of linalool-mediated resistance against Xanthomonas citri subsp. citri and Penicilium italicum in citrus leaves and fruits. Plant Sci.

[46] Shimada T, Endo T, Rodríguez A, Fujii H, Goto S, Matsuura T, Hojo Y, Ikeda Y, Mori IC. Fujikawa T ectopic accumulation of linalool confers resistance to *Xanthomonas citri* subsp. *citri* in transgenic sweet orange plants. Tree Physiol 2017(5):654. 10.1093/treephys/tpw134 28131994

[47] Dorman H, Deans SG (2010). Antimicrobial agents from plants: antibacterial activity of plant volatile oils. J Aappl Microbiol.

[48] Park SN, Lim YK, Freire MO, Cho E, Jin D (2012). Kook JK Antimicrobial effect of linalool and alpha-terpineol against periodontopathic and cariogenic bacteria. Anaerobe.

[49] Yamasaki Y, Kunoh H, Yamamoto H, Akimitsu K (2007). Biological roles of monoterpene volatiles derived from rough lemon (*Citrus jambhiri* Lush) in citrus defense. J Gen Plant Pathol.

[50] Droby S, Eick A, Macarisin D, Cohen L, Rafael G, Stange R, McColum G, Dudai N, Nasser A, Wisniewski M (2008). Role of citrus volatiles in host recognition, germination and growth of *Penicillium digitatum* and *Penicillium italicum*. Postharvest Biol Tec.

[51] Alves S, Duarte A, Sousa S, Domingues FC (2016). Study of the major essential oil compounds of *Coriandrum sativum* against *Acinetobacter baumannii* and the effect of linalool on adhesion, biofilms and quorum sensing. Biofouling.

[52] Shimada T, Endo T, Fujii H, Rodriguez A, Yoshioka T, Pena L, Omura M (2021). Biological and molecular characterization of linalool-mediated field resistance against *Xanthomonas citri* subsp. *citri* in citrus trees. Tree Physiol.

[53] De Carvalho SA, de Carvalho Nunes WM, Belasque J, Machado MA, Croce-Filho J, Bock CH (2015). Abdo Z comparison of resistance to asiatic citrus canker among different genotypes of citrus in a long-term canker-resistance field screening experiment in Brazil. Plant Dis.

[54] Matsumura M, Nomoto M, Itaya T, Aratani Y, Iwamoto M, Matsuura T, Hayashi Y, Mori T, Skelly MJ (2022). Yamamoto YY Mechanosensory trichome cells evoke a mechanical stimuli–induced immune response in *Arabidopsis thaliana*. Nat Commun.

[55] Ahmad MM, Rehman SU, Anjum FM, Bajwa EE (2006). Comparative physical examination of various citrus peel essential oils. Int J Agric Biol.

[56] Uji Y, Ozawa R, Shishido H, Taniguchi S, Takabayashi J, Akimitsu K, Gomi K (2015). Isolation of a sesquiterpene synthase expressing in specialized epithelial cells surrounding the secretory cavities in rough lemon (*Citrus jambhiri*). J Plant Physiol.

[57] Miller B, Madilao LL, Ralph S, Bohlmann J (2005). Insect-induced conifer defense. White pine weevil and methyl jasmonate induce traumatic resinosis, de novo formed volatile emissions, and accumulation of terpenoid synthase and putative octadecanoid pathway transcripts in Sitka spruce. Plant Physiol.

[58] Zulak KG, Lippert DN, Kuzyk MA, Domanski D, Chou T, Borchers CH, Bohlmann J (2009). Targeted proteomics using selected reaction monitoring reveals the induction of specific terpene synthases in a multi-level study of methyl jasmonate-treated Norway spruce (*Picea abies*). Plant J.

[59] Fang XF, Meng XN, Zhang J, Xia MH, Cao SQ, Tang XF, Fan TT (2021). AtWRKY1 negatively regulates the response of Arabidopsis thaliana to Pst. DC3000. Plant Physiol Bioch.

[60] Logemann E, Birkenbihl RP, Ülker B (2006). Somssich IE An improved method for preparing * Agrobacterium * cells that simplifies the * Arabidopsis * transformation protocol. Plant Methods.

