# Supplementary material for: Promoter characterization of a citrus linalool synthase gene mediating interspecific variation in resistance to a bacterial pathogen
Source: BMC Plant Biol. 2023 Aug 25;23:405. doi: 10.1186/s12870-023-04413-6 (PMC10463377; doi:10.1186/s12870-023-04413-6)
Supplement: Supplementary file 1 — Supplementary Material 1 [file 12870_2023_4413_MOESM1_ESM.docx]

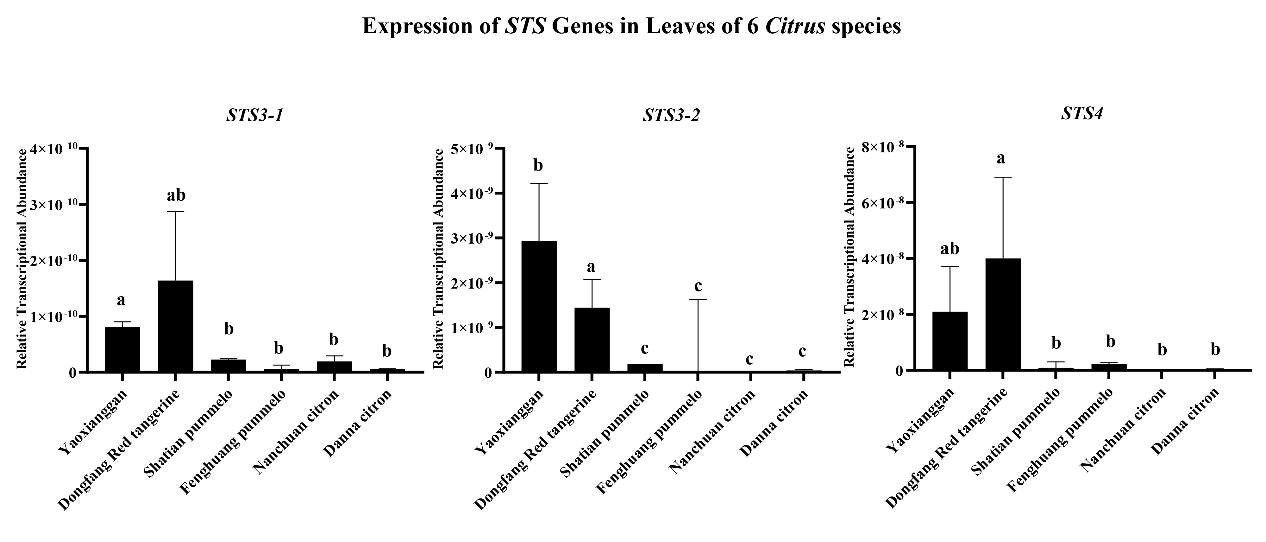


**Fig. S1** Expression analysis of linalool synthase genes in leaves of six *Citrus* varieties (n=3, *p* < 0.05, ANOVA).


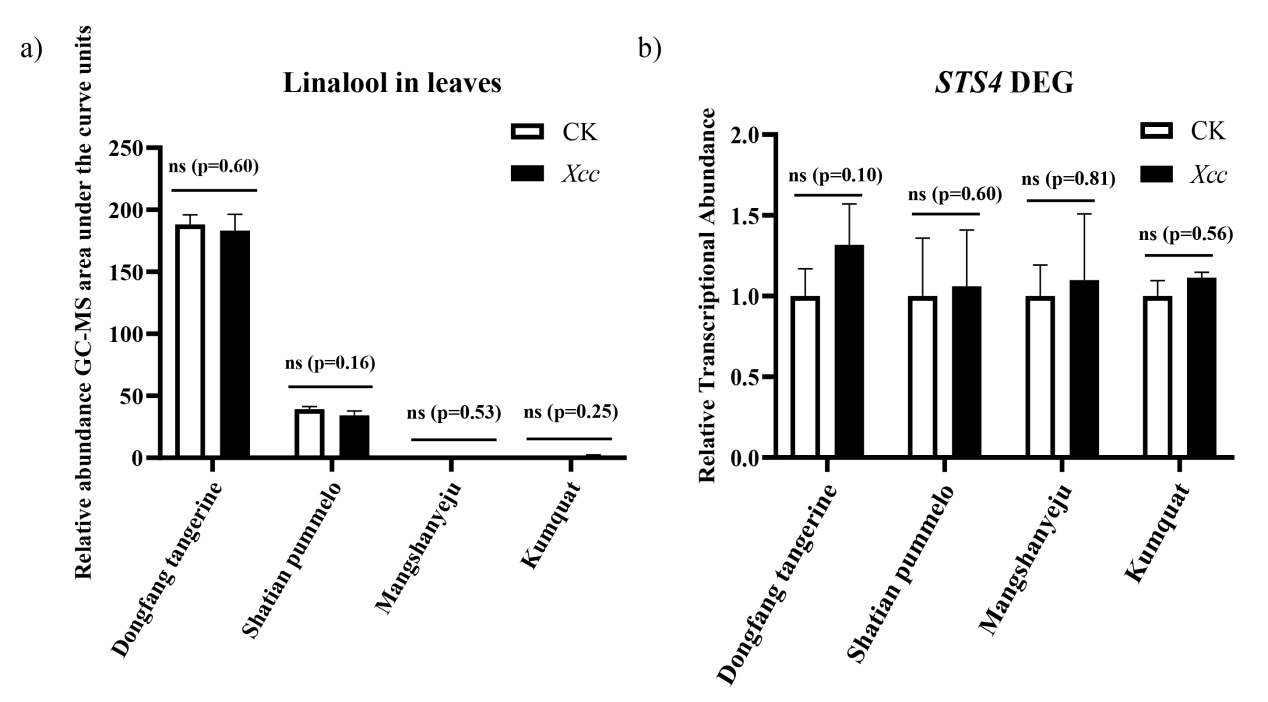
**Fig. S2** *Xcc* infection does not affect the linalool content (a) and transcript abundance of *STS4* gene (b) in different citrus varieties (n=3, *t*-test).

**Table S1. *cis*-elements identified in p*CgSTS4* and *pCrSTS4***

| *cis*-element | *cis*-element number | | Core sequence | Function |
| --- | --- | --- | --- | --- |
|  | *CgSTS4* | *CrSTS4* |  |  |
| AAGAA-motif | 1 | 0 | GAAAGAA | abscisic acid response element |
| ABRE | 4 | 4 | CACGTG | abscisic acid responsiveness |
| ABRE3a | 2 | 2 | TACGTG | abscisic acid responsiveness |
| ABRE4 | 2 | 2 | CACGTA | abscisic acid responsiveness |
| AE-box | 3 | 3 | AGAAACAA | light responsive element |
| ARE | 3 | 2 | AAACCA | anaerobic induction |
| AT~TATA-box | 9 | 7 | TATATA | AT-rich DNA binding protein (ATBP-1) |
| AT-rich element | 2 | 2 | ATAGAAATCAA | Determine transcription initiation |
| Box 4 | 4 | 3 | ATTAAT | light responsive element |
| CAAT-box | 37 | 34 | CAAAT | promoter and enhancer regions |
| ERE | 2 | 3 | ATTTTAAA | ethylene-responsive element |
| F-box | 1 | 1 | CTATTCTCATT | gibberellin-responsive element |
| GATA-motif | 1 | 0 | AAGATAAGATT | light responsive element |
| G-box | 5 | 5 | CACGTG | light responsive element |
| GC-motif | 1 | 1 | CCCCCG | anoxic specific inducibility |
| GCN4-motif | 1 | 1 | TGAGTCA | endosperm expression |
| GT1-motif | 1 | 1 | GGTTAA | light responsive element |
| MBS | 1 | 1 | CAACTG | drought-inducibility |
| MRE | 1 | 1 | AACCTAA | light responsive element |
| MYB | 5 | 3 | CAACCA | Myb binding site |
| Myb | 3 | 1 | TAACTG | Myb binding site |
| MYC | 5 | 3 | CATTTG | Myc binding site |
| Myc | 7 | 5 | TCTCTTA | Myc binding site |
| P-box | 1 | 1 | CCTTTTG | gibberellin-responsive element |
| STRE | 3 | 2 | AGGGG | stress response element |
| TATA-box | 75 | 70 | TATA/ATATAA | core promoter element around -30 of transcription start |
| TCCC-motif | 1 | 1 | TCTCCCT | light responsive element |
| TC-rich repeats | 1 | 1 | GTTTTCTTAC | stress response element |
| WUN-motif | 1 | 2 | AAATTACT | Wounding response element |

**Table S2. Sequence of primers used in this study**

| Primer Name | Primer **Sequence（5’-3’）** |
| --- | --- |
| CrSTS4-CDS-F | ATGTCGTTTCCGGTTTCAGCC |
| CrSTS4-CDS-R | CTAGTCTTCAAAGAAAACAGGATCCTTAAGCA |
| CgSTS4-CDS-F | ATGTCGTTTCAAGTTTCAGCCTCTC |
| CgSTS4-CDS-F | CTAGTCTTCAAAGAAAACAGGATCCTTAAGCA |
| STS3-1-qPCR-F | TTCGGCTCTGGGATGACTTG |
| STS3-1-qPCR-R | TGGGCAGAGGCATTCTTTGT |
| STS3-2-qPCR-F | ACAGCAACCCACCCATTGTA |
| STS3-2-qPCR-R | CAGCCGTTAAGTCTCGGTGT |
| STS4-qPCR-F | ATGCCGTTGCACTACTTTC |
| STS4-qPCR-R | AGGCCACATGTCCTCTCTGT |
| CitActin-F | CATCCCTCAGCACCTTCC |
| CitActin-R | CCAACCTTAGCACTTCTCC |
| Pcam-CrSTS4-pro-F | tagatctccaggatccAGAAGCCCCCGCTAATATTATGTTCT |
| Pcam-CrSTS4-pro-R | ttaaagcagggaattcGTCCTTAATTTCTATTGCTTGCTAAAGGCAT |
| Pcam-CgSTS4-pro-F | tagatctccaggatccAGAAGCCCCCGCTAATATTATGTTCT |
| Pcam-CgSTS4-pro-R | ttaaagcagggaattcGTCCTTAATTTCTATTGCTTGCTAAAGGCAT |
| CrSTS4-transgenic-Test-F | TGTCCAACCAAAAGCGAGAGA |
| CrSTS4-transgenic-Test-R | CACATTGCAAGATCTCCCTCC |
| CgSTS4-transgenic-Test-F | GTTTTCACTTGTCGTGTCGCT |
| CgSTS4-transgenic-Test-R | CCACACATTGCAAGATCTCCCT |
| GUS-qPCR-F | CGAAGCGAGCAATGTGATGG |
| GUS-qPCR-R | GATCCGCAAGACGCATCAAC |
